# Micro-CT to Document the Coffee Bean Weevil, *Araecerus fasciculatus* (Coleoptera: Anthribidae), Inside Field-Collected Coffee Berries (*Coffea canephora*)

**DOI:** 10.3390/insects9030100

**Published:** 2018-08-14

**Authors:** Ignacio Alba-Alejandre, Javier Alba-Tercedor, Fernando E. Vega

**Affiliations:** 1 Department of Zoology, Faculty of Sciences, University of Granada, Campus de Fuentenueva, 18071 Granada, Spain; ignacioalba@gmail.com (I.A.-A.); jalba@ugr.es (J.A.-T.); 2 Sustainable Perennial Crops Laboratory, United States Department of Agriculture, Agricultural Research Service, Beltsville, MD 20705, USA

**Keywords:** coffee quality, insect biology, losses, stored coffee, stored product pest

## Abstract

The coffee bean weevil, *Araecerus fasciculatus* (De Geer) (Coleoptera: Anthribidae), is a cosmopolitan insect with >100 hosts, and has been reported as a pest of stored coffee. During a study involving the coffee berry borer, we observed coffee bean weevils emerging from field-collected coffee berries and used micro-computerized tomography (micro-CT) scans to observe the insect inside the berry. Two eggs had eclosed inside the berry, resulting in observations of a newly eclosed adult beetle and a 5th instar larva, each feeding on one of the two seeds. This is the first time since 1775, when the insect was first described, that the insect has been observed inside a coffee berry.

## 1. Introduction

The genus *Araecerus* Schönherr comprises ca. 75 species [1], with the coffee bean weevil, *Araecerus fasciculatus* (De Geer) (Coleoptera: Anthribidae), being the most economically important. Chittenden [2,3] coined the name coffee bean weevil and Valentine [1] has published a succinct account on the controversy involving the many different scientific names used for the insect.

The coffee bean weevil is ca. 4–5 mm long [4], has a worldwide distribution, over 100 hosts, and is mostly considered a stored product pest [5,6]. The insect has been occasionally reported as a pest of stored coffee beans, into which females insert an egg 1–2 mm deep, followed by larval consumption of the bean [7]. In Brazil, de Figuereido Jr. [8] reported ca. 30% losses in coffee stored for 6 months, and Abrahão and Bitran [9] reported 20% losses in coffee stored for 9 months. In Colombia, Cabal Concha [7] reported heavy infestation of stored coffee in several locations. Depending on temperatures, there could be 8–10 insect generations per year in stored green coffee [8].

**Figure 1:**
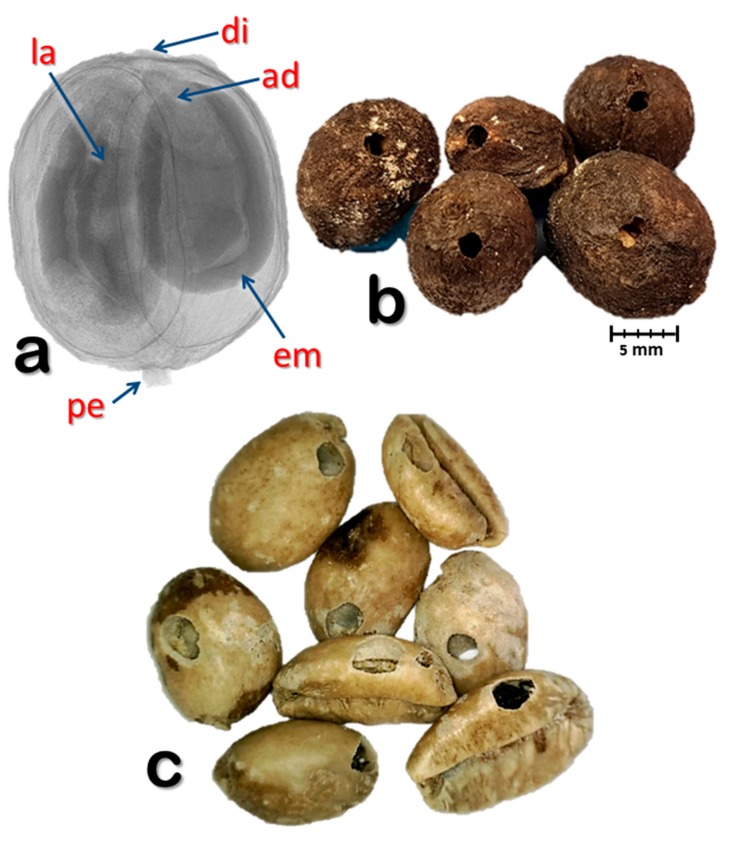
X-ray image of a coffee bean weevil-infested coffee berry (**a**). Infested coffee berries showing the adult weevil exit hole next to the disc (**b**). Green coffee beans (*Coffea liberica*) from British Guiana damaged by the coffee bean weevil (**c**). Abbreviations: ad = adult; di = disc (style remnant); em = coffee embryo; la = larva; pe = pedicel.

In addition to the losses caused by the insect, green coffee exhibiting insect damage (Figure 1c) is considered a defect and will negatively impact on grading and quality [10]. Furthermore, insect damage could also result in the presence of insect fragments. Locatelli and Viganò [11] determined the presence of insect fragments in 44 *C. arabica* and 27 *C. canephora* green coffee beans samples in Italy and found that 23% and 15% of the samples, respectively, contained coffee bean weevil fragments.

Even though it has been reported that the coffee bean weevil only attacks stored green coffee beans [12,13], the insect also attacks coffee berries in the field. For example, referring to the coffee bean weevil and *Coffea arabica*, Chittenden [3] stated, “the raw berry of which it also infests.” In Brazil, Autuori [4] has reported that females oviposit up to six eggs inside berries, but only one, and rarely two, eclose. The larva initially feeds on the pulp or on mucilage between the two seeds, followed by penetration into the seed and consumption of the coffee bean [4]. Also in Brazil, da Costa Lima [14] mentions that the insect can be found in coffee plantations, although in small numbers, and Abrahão and Bitran [9] reported 4.2% infestation in the field. The insect has also been reported in coffee berries in the field in Togo [15] and Ghana [16]. Sekhar [17] reported infestations in the field in India, which is incorrect based on the following statement: “Fruits infested by the weevil show circular holes, 0.5–1.0 mm. in diameter.” The size of these holes corresponds to the coffee berry borer (see Results and Discussion). The Directoria de Agricultura [18] in Brazil recommended that “When the insect attacks the coffee fruit still on the tree, the fruit should be harvested and burned or disinfected, because otherwise, it will be impossible to avoid that the larvae, which are inside the beans, reach the adult stage”.

As part of our studies aimed at learning more about the behavior of the coffee berry borer (*Hypothenemus hampei* (Ferrari); Coleoptera: Curculionidae: Scolytinae) inside the berry [19], we observed coffee bean weevils emerging from coffee berries collected in the field. We report on the use of micro-computerized tomography (micro-CT) scans to observe and record coffee bean weevils inside coffee berries collected in a coffee plantation in Vietnam.

## 2. Materials and Methods

### 2.1. Coffee Berries

Fifty coffee berries (*Coffea canephora* Pierre ex. A. Froehner; Rubiaceae) which were red or starting to turn red, were randomly collected from several coffee plants by the second author in November 2017 at the *Me Linh Coffee Garden* plantation in southern Vietnam (11°53′57.39′′ N, 108°20′51.16′′ E; 1043 m above sea level). The berries were kept at ambient temperature in Petri dishes containing moistened filter paper in the laboratory at the Department of Zoology, University of Granada, Spain. While examining the berries 63 days after they were collected, we noticed the presence of coffee bean weevils in the Petri dish (Supplementary Video S1). To determine if berries were still infested with weevils, we visualized the internal parts of the berry (Figure 1a) using X-rays produced by a high-resolution micro-CT system (see below), until movement was detected. The first berry in which movement was detected was used for the micro-CT study.

### 2.2. Micro-CT Scans

**Figure 2:**
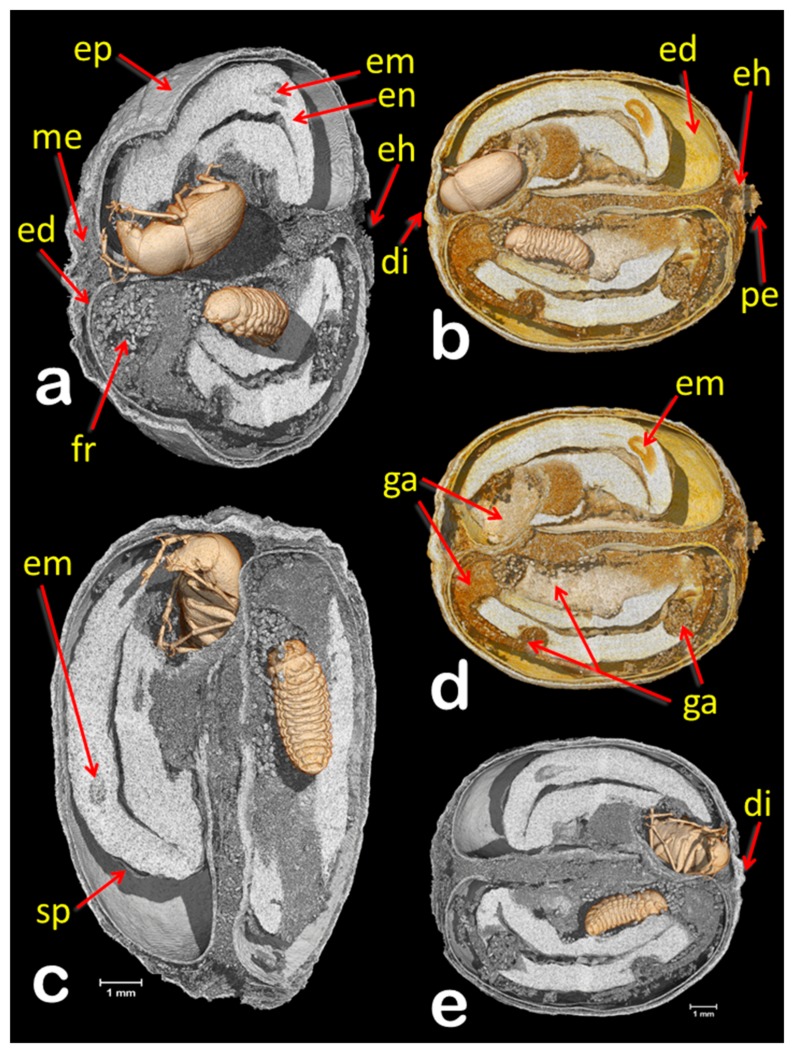
Micro-CT volume rendering images of a coffee berry infested with a female adult coffee bean weevil and a 5th instar larva that have been separately segmented to unveil their location inside the berry. Latero-apical cut view (**a**). Lateral cut view (**b**). Lateral cut views, at two perpendicular cut planes (**c**). Same view shown in “(**b**)”, but the insects have been eliminated with software to enhance the galleries (**d**). Lateral cut view from side opposite to that shown in “(**b**)” (**e**). Abbreviations: di = disc (style remnant); ed = endocarp (parchment); eh = entrance hole; em = coffee embryo; en = endosperm (seed); ep = epicarp (outer skin); fr = frass; ga = galleries; me = mesocarp (mucilage); pe = pedicel; sp = spermoderm (silverskin).

**Figure 3:**
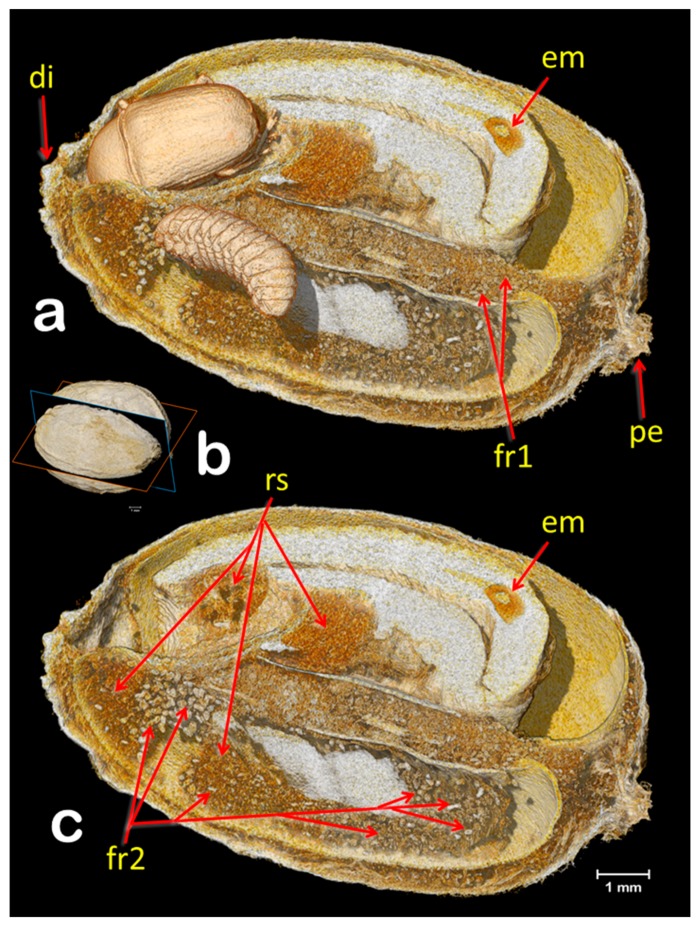
Volume rendering images of a coffee berry infested with the coffee bean weevil (**a**,**c**), sectioned as shown in (**b**). The female adult and 5th instar larva (**a**) have been separately segmented to unveil their location inside the berry. Digital removal of the insects (**c**) allows to see the cavities and galleries filled with frass (fr1, fr2) and rasped seed material (rs). It is possible to distinguish the older galleries occupied during the time the larva was younger and smaller because they are filled with a smaller frass size (ca. 175 μm long; fr1). Newer galleries have a larger frass size (ca. 290 μm long; fr2). Abbreviations: di = disc (style remnant); em = coffee embryo; pe = pedicel.

A coffee bean weevil-infested coffee berry was mounted on a piece of Basotect^®^ (low weight melamine resin foam; BASF, Schwarzheide, Germany), inside a plastic container. Basotect^®^ has a very low density that makes it transparent to X-rays, thus allowing the material to be digitally removed during the segmentation process [20]. To avoid insect movement during the scans, insects were killed by adding several drops of ethyl acetate to the melamine foam followed by closing the container. Scans were initiated 30 min later with a Bruker SkyScan 1172 high-resolution desk-top microtomograph (Bruker, Kontich, Belgium) upgraded with a Hamamatsu L7902 100/250 X-ray source and a Ximea (SHT) 11 megapixels camera (Ximea GmbH, Münster, Germany). The scanning parameters were as follows: isotropic voxel size = 5.96 µm per pixel; voltage = 48 KV, current = 124 µA; image rotation step = 0.2°; 360° of rotation scan and an Al 0.5 mm filter, resulting in two connected scans and 2400 X-ray raw images. The most recent version of the Bruker micro-CT’s Skyscan software (NRecon, DataViewer, CTAnalyser) was used for primary reconstructions and the “cleaning” process to obtain the datasets of “slices” as described by Alba-Tercedor [21]. Amira’s Software 6.4.0 [22] was used to obtain volume rendering reconstructions images in Figure 2 and Figure 3, to make the Supplementary Video S2, and to measure the width of the larval cephalic capsule and the length of frass.

The macrophotograph of coffee berries shown in Figure 1b and the Supplementary Video S1 were obtained using a Samsung Galaxy Note8 smartphone. The macrophotograph of the coffee bean weevil was taken with an AxioZoom V16 zoom microscopy system (Carl Zeiss Microscopy LLC, Thornwood, NY, USA). The images were observed using a 1.0x/0.25 NA or 2.3x/0.25 NA Plan Neofluar^®^ objective. LED lighting was used for brightfield imaging and a Zeiss AxioCam HRc color camera (Carl Zeiss Light Microscopy, Gottingen, Germany) was used to capture the images. ZEN imaging software (Carl Zeiss Microscopy LLC, Thornwood, NY, USA) was used to capture 60–75 z-stack images using extended depth of focus to produce 2D images. The macrophotograph of *Ptinus sensu stricto* was taken with a Samsung Galaxy Note8 smartphone connected to the ocular of a Motic SMZ168 Stereo Zoom microscope (MoticEurope S.L.U., Barcelona, Spain).

## 3. Results and Discussion

**Figure 4:**
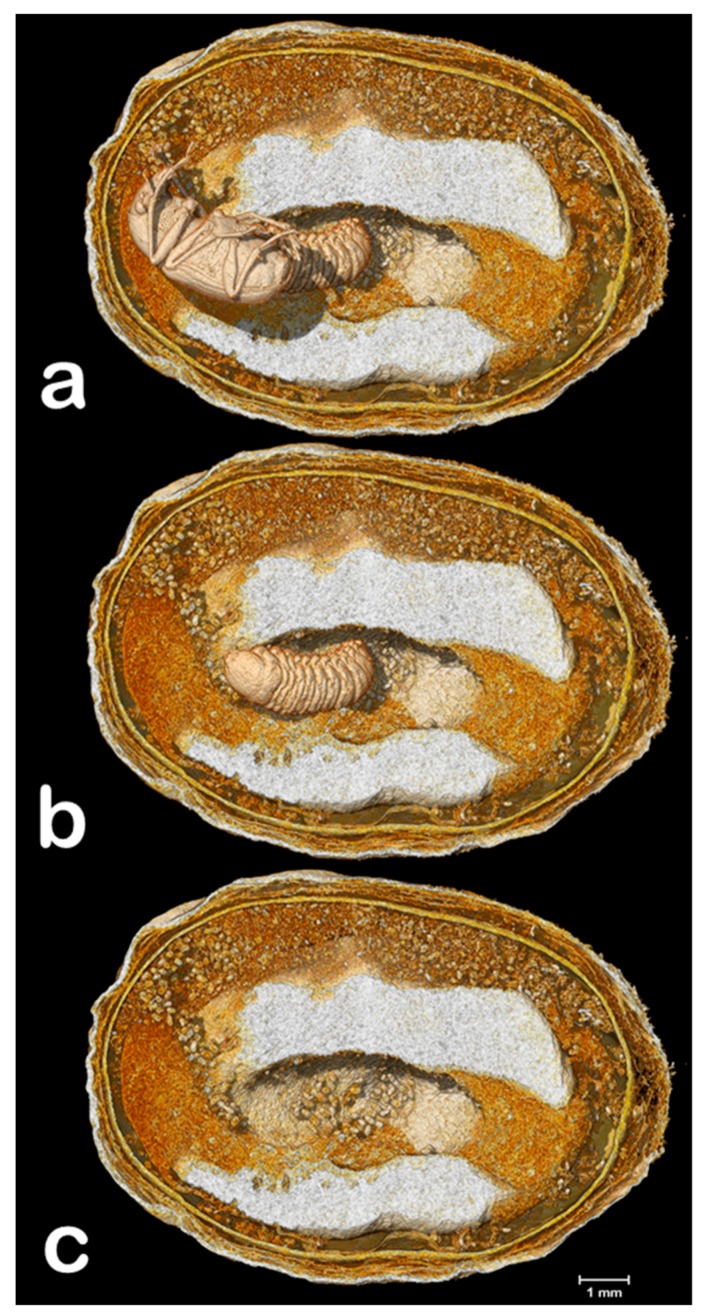
Volume rendering images of a coffee berry infested with the coffee bean weevil, sectioned in a perpendicular plane in relation to Figure 2b,d. Images show galleries partially filled with frass and rasped seed material. The position of the insects is shown in (**a**). The adult female was digitally removed, leaving the 5th instar larvae (**b**). Both insects have been digitally removed (**c**).

Based on the presence of emergence holes (Figure 1b) 63 days after the berries were collected, six out of 50 berries (12%) were infested with the coffee bean weevil. All exit holes were located next to the disc. Five berries were photographed (Figure 1b); the sixth berry is the one used in the study, which did not yet have an emergence hole, although both the female adult and 5th instar larva (Figure 2, Figure 3 and Figure 4) have their anterior part positioned towards the disc.

**Figure 5:**
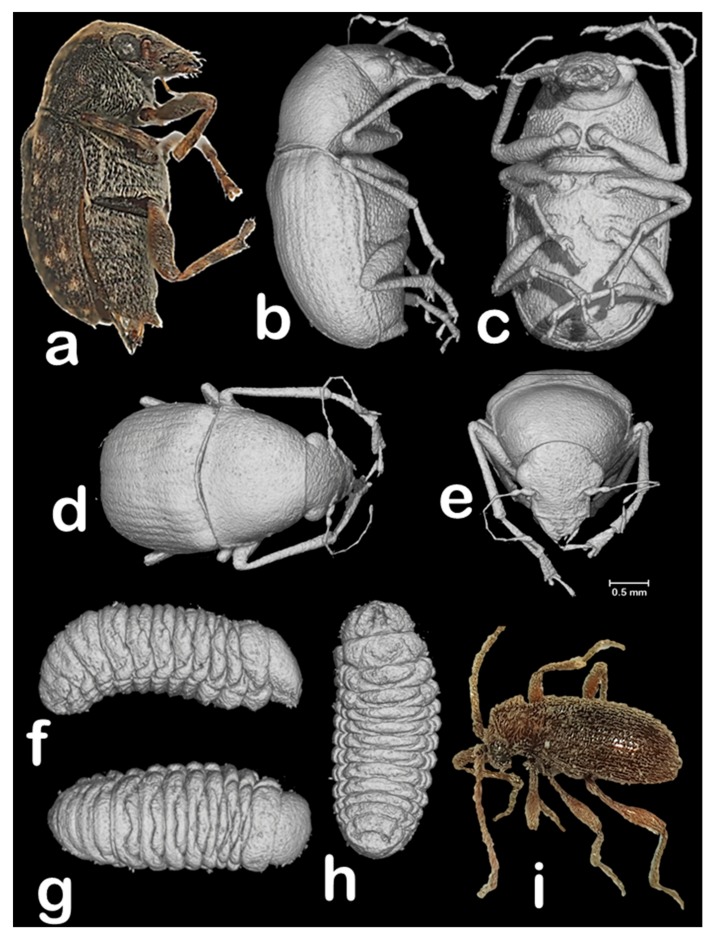
Female adult and larva of the coffee bean weevil (**a**–**h**), and macrophotography of a male of *Ptinus sensu stricto* (**i**). Macrophotography (**a**) and micro-CT volume rendering images (**b**–**h**) of the same coffee bean weevil specimens inside the coffee berry shown in Figure 2, but digitally extracted from the berry and visualized with Amira software [22]. Female adult (**a**–**e**) and a 5th instar larva (**f**–**h**). Lateral views (**a**,**b**,**f**); ventral views (**c**,**h**); antero-dorsal view (**d**); dorsal view (**g**); and frontal view (**e**).

The coffee bean weevil has five instars that can be identified using the width of the cephalic capsule [7,23]; therefore, the larva shown in Figure 2, Figure 3 and Figure 4 is a 5th instar (0.903 mm wide). Figure 2 reveals three interesting findings: (1) two eggs had eclosed, which according to Autuori [4] is rare; (2) both seeds are being consumed; and (3) there is a partial entrance hole. It is worth noting that we also collected a male of *Ptinus sensu stricto* (Ptinidae) that had emerged from a coffee berry (Figure 5i). *Ptinus tectus* has been reported on stored coffee [24].

As mentioned above, female coffee bean weevils oviposit inside the berry. Therefore, the partial entrance hole in the berry (Figure 2) posed a conundrum. The entrance hole is much too small (ca. 0.95 mm diam.) for a coffee bean weevil, whose width is 2–3 mm [4] and whose emergence hole can be up to 3 mm diam. [14]. Despite being close to the petiole and not on the disc, which is where the coffee berry borer colonizing female usually bores into the berry, the partial hole appears to have been bored by a coffee berry borer, whose entrance holes range from 0.6–0.8 mm [25] to 1 mm diam [26].

It is possible that the coffee bean weevil oviposited in the partially bored entrance hole, where one egg eclosed. The first instar larvae then bored into the berry, based on the connection between the partial entrance hole and a gallery filled with ca. 175 μm long frass and rasped seed material (Figure 2, Figure 3 and Figure 4; according to Autuori [4], larvae gnaw more than they eat), and as the gallery progresses away from the partial entrance hole, the length of the frass increases to ca. 290 μm (Figure 3 and Figure 4), which indicates an older larval instar. Cabal Concha [7] mentions that 4th and 5th instars are more active and voracious than earlier instars, and that not all the material they gnaw is consumed (thus supporting Autuori’s observation [4]) and instead, is accumulated. At times of highest heat intensity, they spread it around their body which is hypothesized to (1) create a barrier with the seed wall thus reducing the heat intensity and (2) serve as a defense against parasitoids and predators trying to reach the insect [7].

## 4. Conclusions

Even though *A. fasciculatus* was first reported by Charles De Geer in 1775 as *Curculio fasciculatus* [27], this is the first time in the intervening 242 years we have been able to “freeze” the activity of the insect inside a coffee berry and study it in detail using modern micro-CT techniques. This study reveals interesting aspects of the biology of the coffee bean weevil inside the coffee berry.

## Supplementary Materials

The following are available online. Supplementary Video S1: Coffee bean weevil walking over coffee berries inside Petri dish: https://drive.google.com/open?id=1_tEYfNdTy9qHKbOEbXwLs0hPZCXMb4lF & Supplementary Video S2: Micro-CT volume rendering of a coffee bean weevil adult and 5th larval instar inside a coffee berry: https://drive.google.com/open?id=1WWM9ABL7e6gkFOBZBroKloAbf6taOO6g.
